# Up-regulated extracellular matrix components and inflammatory chemokines may impair the regeneration of cholestatic liver

**DOI:** 10.1038/srep26540

**Published:** 2016-05-26

**Authors:** Shuai Zhang, Tao-Sheng Li, Akihiko Soyama, Takayuki Tanaka, Chen Yan, Yusuke Sakai, Masaaki Hidaka, Ayaka Kinoshita, Koji Natsuda, Mio Fujii, Tota Kugiyama, Zhassulan Baimakhanov, Tamotsu Kuroki, Weili Gu, Susumu Eguchi

**Affiliations:** 1 Department of Surgery, Nagasaki University Graduate School of Biomedical Sciences, Japan; 2 Department of Hepatopancreatobiliary Surgery, Guangzhou First People’s Hospital, Guangzhou Medical University, Guangzhou Digestive Disease Center, Guangzhou 510180, China; 3 Department of Stem Cell Biology, Atomic Bomb Disease Institute, Nagasaki University, 1-12-4 Sakamoto, Nagasaki 852-8523, Japan

## Abstract

Although the healthy liver is known to have high regenerative potential, poor liver regeneration under pathological conditions remains a substantial problem. We investigated the key molecules that impair the regeneration of cholestatic liver. C57BL/6 mice were randomly subjected to partial hepatectomy and bile duct ligation (PH+BDL group, n = 16), partial hepatectomy only (PH group, n = 16), or sham operation (Sham group, n = 16). The liver sizes and histological findings were similar in the PH and sham groups 14 days after operation. However, compared with those in the sham group, the livers in mice in the PH+BDL group had a smaller size, a lower cell proliferative activity, and more fibrotic tissue 14 days after the operation, suggesting the insufficient regeneration of the cholestatic liver. Pathway-focused array analysis showed that many genes were up- or down-regulated over 1.5-fold in both PH+BDL and PH groups at 1, 3, 7, and 14 days after treatment. Interestingly, more genes that were functionally related to the extracellular matrix and inflammatory chemokines were found in the PH+BDL group than in the PH group at 7 and 14 days after treatment. Our data suggest that up-regulated extracellular matrix components and inflammatory chemokines may impair the regeneration of cholestatic liver.

The volume and function of a healthy liver can recover to nearly normal levels within weeks after either partial hepatectomy or various injuries, suggesting that the liver has a high regenerative capacity^1^,^2^,^3^. However, many pathological factors, such as viral infections and cholestatic disorders, significantly impair this regeneration capacity, which leads to insufficient liver regeneration following surgical resection, and an increased risk of postoperative complications^4^. Stem cell therapy has been clinically applied to improve liver regeneration but is currently limited by its marginal efficiency^5^,^6^. Therefore, a need remains to understand the mechanisms underlying poor liver regeneration under pathological conditions, because the identification of key factor(s) may help to identify a new efficient strategy for enhancing liver regeneration and reducing complications after liver surgery.

Cholestasis is one of the most common complications in patients who have undergone partial hepatectomy. It is also generally known that cholestasis destroys the normal liver architecture, causes liver fibrosis, and ultimately leads to hepatic dysfunction and portal hypertension^7^. The accumulation of bile acids in the cholestatic liver may directly lead to hepatic damage^8^ because the exposure of hepatocytes to high concentrations of bile acid has been demonstrated to induce cell death^9^. However, it is not fully understood how cholestatic disorders impair the liver’s regenerative capacity.

Complex factors, including the “seed” (i.e., hepatocytes and hepatic progenitor cells) and “soil” (i.e., extracellular matrix and adhesion molecules, cytokines/chemokines and growth factors) are well known to regulate the regeneration process of the liver after injury. Previous studies have reported that some cytokines play important roles in perioperative complications and are involved in the pathogenesis of cholestatic liver disease^10^,^11^,^12^. Gäbele *et al.* have further demonstrated that tumour necrosis factor (TNF)-α is crucial for liver injury and liver fibrosis that is induced by cholestasis in mice^13^.

In the present study, we performed partial hepatectomy and bile duct ligation in mice, and then compared the regenerative potency between healthy and cholestatic livers after partial hepatectomy. We also analysed the changes of the gene expression profiles over time after partial hepatectomy in either healthy or cholestatic livers by using pathway-focused PCR arrays, which allowed us to further identify the key molecule(s) for liver regeneration.

## Results

### Insufficient regeneration of cholestatic liver after partial hepatectomy

**Figure 1:**
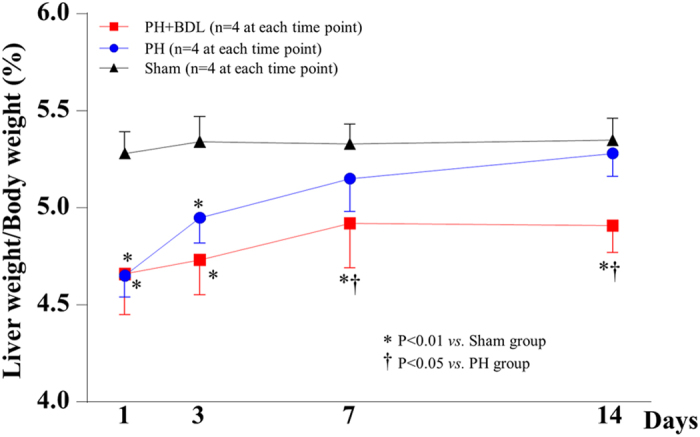
The changes in liver size after treatment over time. The ratio of liver weight to body weight of mice at 1, 3, 7, and 14 days after a sham operation (Sham group), partial hepatectomy alone (PH group), and partial hepatectomy and bile duct ligation (PH+BDL group).

By calculating the ratio of liver weight to body weight (LW/BW) for each mouse, we found that the LW/BW ratios were significantly lower in the PH and PH+BDL groups compared with the sham group at 1 and 3 days after treatment (p < 0.01, Fig. 1). The LW/BW ratio in the PH group increased with time and resulted in a liver size that was comparable to that in the sham group within 14 days after treatment (Fig. 1). However, the LW/BW ratio in the PH+BDL group increased slowly and was still significantly lower 14 days after partially hepatectomy, indicating an insufficient regeneration in the cholestatic liver.

### Poor cell proliferation and fibrotic change of cholestatic liver after partial hepatectomy

**Figure 2:**
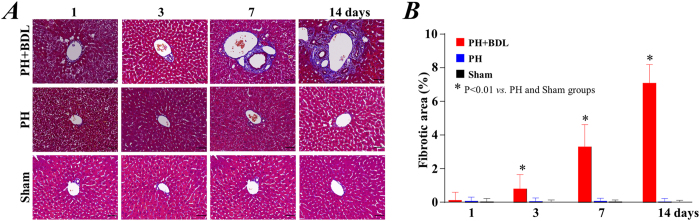
Histological findings and hepatic fibrosis. (**A**) Representative images of Masson’s staining for hepatic fibrosis at 1, 3, 7, and 14 days after treatments. (**B**) Quantitative data on the fibrotic area within the liver tissue after treatments in each group. Abbreviations: PH+BDL, partial hepatectomy and bile duct ligation; PH, partial hepatectomy only; Sham, sham operation.

Masson’s trichrome staining was performed to evaluate the fibrotic changes in the liver. For the mice in the PH+BDL group, the liver exhibited positive staining indicating fibrosis, and the fibrotic area increased over time, reaching the highest level 14 days after operation (Fig. 2A). In contrast, we found few fibrotic areas in the livers of mice from the PH and sham groups (Fig. 2A), confirming the near-complete regeneration of a healthy liver within 2 weeks after 70% partial hepatectomy. Quantitative measurements showed that the fibrotic area in the PH+BDL group was significantly higher than that in the PH and sham groups at 3, 7, and 14 days after operation (Fig. 2B).

**Figure 3:**
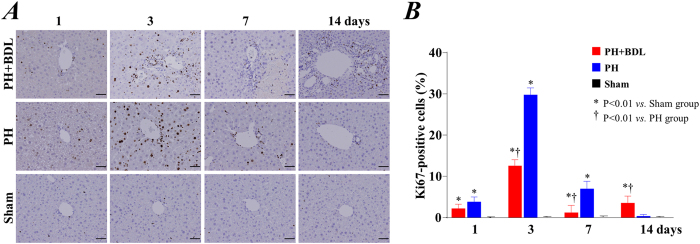
Cell proliferation activity. (**A**) Representative images of immunostaining for the nuclear proliferating antigen Ki67 at 1, 3, 7, and 14 days after treatment. (**B**) Quantitative data showing the positive expression of Ki67 within the liver tissue after treatments in each group. Abbreviations: PH+BDL, partial hepatectomy and bile duct ligation; PH, partial hepatectomy only; Sham, sham operation.

We also evaluated cell proliferative activity by immunostaining with the antigen Ki67, one of the most commonly used markers for cell proliferation. Enhancement of Ki67 expression was uniformly observed in all of the areas of liver in the PH and PH+BDL groups at 1 and 3 days, suggesting the initiation of the regenerative process in the liver soon after partial hepatectomy (Fig. 3A). Quantitative data showed that the expression of Ki67 was significantly lower in the PH+BDL group than in the PH group at 3 and 7 days after operation (Fig. 3B).

**Figure 4:**
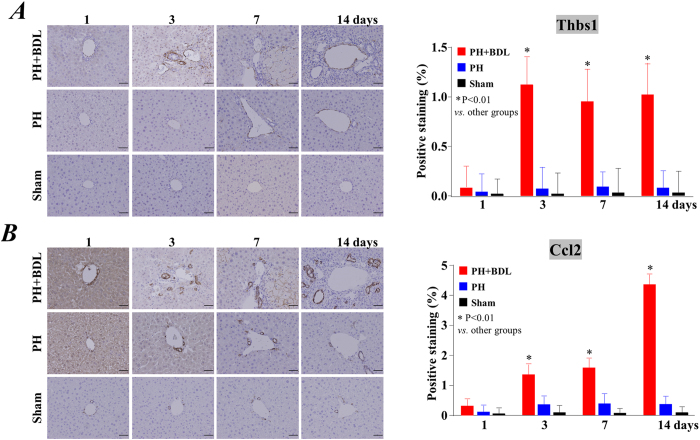
The expression of thrombospondin 1 and the chemokine (C-C motif) ligand 2. Representative images show immunostaining for thrombospondin 1 (**A**) and the chemokine (C-C motif) ligand 2 (**B**) in the liver of mice at 1, 3, 7, and 14 days after treatment (left panels). Quantitative data of positive staining was also shown as bar graphs (right panels). Abbreviations: PH+BDL, partial hepatectomy and bile duct ligation; PH, partial hepatectomy only; Sham, sham operation.

In addition, we also used immunostaining to evaluate the expression of thrombospondin 1 (*Thbs1*) and chemokine (C-C motif) ligand 2 (*Ccl2*), two factors that are known to contribute to inflammation and hepatic fibrosis (Fig. 4). Representative images in Fig. 4 show the enhanced expression of Thbs1 (Fig. 4A) and Ccl2 (Fig. 4B) in the liver of the PH+BDL group at 3 days after the operation. Although we selected typical histological images around the periportal area, there were no observable differences in positive staining among the different areas/zones of the liver. Quantitative data showed that the expression of Thbs1and Ccl2 was significantly higher in the PH+BDL group than the PH group and the sham groups at 3, 7 and 14 days after operation (Right bar graphs in Fig. 4). All of these results indicate enhanced inflammation and impaired regenerative capacity in the cholestatic liver after partial hepatectomy.

### Changes in gene expression levels after partial hepatectomy

**Figure 5:**
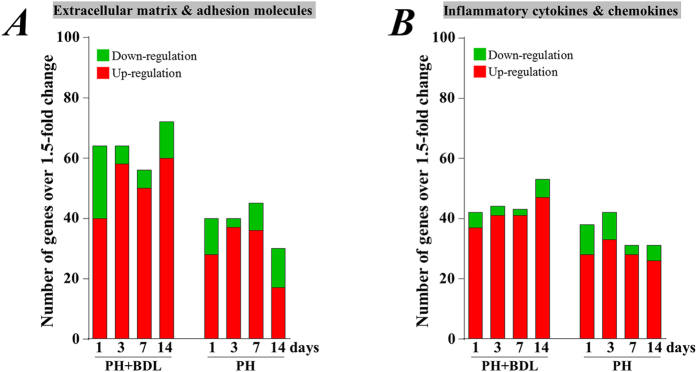
Pathway-focused PCR array analysis of gene expression. (**A**) Several genes associated with extracellular matrix and adhesion molecules were up- and down-regulated by more than 1.5-fold in the liver tissue of mice at 1, 3, 7, and 14 days after partial hepatectomy and bile duct ligation (PH+BDL group) or partial hepatectomy alone (PH group). (**B**) Several genes associated with inflammatory cytokines and chemokines were up- and down-regulated by more than 1.5-fold in the liver tissue of mice at 1, 3, 7, and 14 days after partial hepatectomy and bile duct ligation (PH+BDL group) or partial hepatectomy alone (PH group).

Pathway-focused PCR array analysis showed that a number of genes were up- and down-regulated in liver tissue 1, 3, 7, and 14 days after partial hepatectomy in mice in the PH+BDL and PH groups, as compared with the healthy liver tissue from mice in the sham group. Among the 168 genes that were included in the two pathway arrays that focused on chemokines/cytokines and ECM/adhesion molecules, we detected several genes that were up- or down-regulated by more than 1.5-fold at 1, 3, 7, and 14 days in the PH group (Fig. 5). However, even higher numbers of genes were up- or down-regulated by more than 1.5-fold at 1, 3, 7, and 14 days in the PH+BDL group (Fig. 5). Furthermore, our results showed that the number of genes exhibiting a more than 1.5-fold change tended to decrease at 7 and 14 days in the PH group but were increased even at 14 days in the PH+BDL group (Fig. 5).

**Figure 6:**
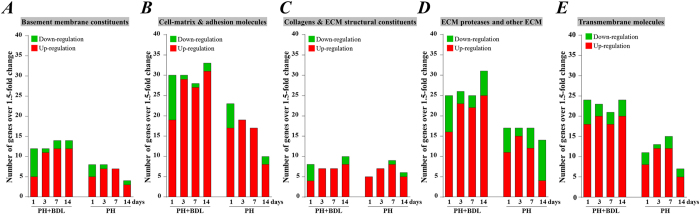
Functional categorization of genes that were included in the mouse extracellular matrix and adhesion molecule PCR array. Based on the biological functions, these genes were categorized into basement membrane constituents (**A**), cell-matrix and adhesion molecules (**B**), collagens and ECM structural constituents (**C**), ECM proteases and other ECM molecules (**D**), and transmembrane molecules (**E**). We counted the number of genes that were up- and down-regulated by more than 1.5-fold in the liver tissue of mice at 1, 3, 7, and 14 days after partial hepatectomy and bile duct ligation (PH+BDL group) or partial hepatectomy alone (PH group).

We further categorized these genes according to their biological functions. Among the genes included in the ECM and adhesion molecule PCR array, we observed that many genes were up-regulated in both the PH and PH+BDL groups (Fig. 6). Interestingly, the numbers of genes that were functionally related to the basement membrane constituents (Fig. 6A), the cell matrix & adhesion molecules (Fig. 6B), ECM proteinase (Fig. 6D), and transmembrane molecules (Fig. 6E) were dramatically decreased 14 days after operation in the PH group but remained stable or even tended to increase with time in the PH+BDL group. Moreover, several genes that were functionally related to collagens and ECM structural constituents (Fig. 6C) were down-regulated in the PH+BDL group, but no genes were down-regulated in the PH group at day 1 after operation.

**Figure 7:**
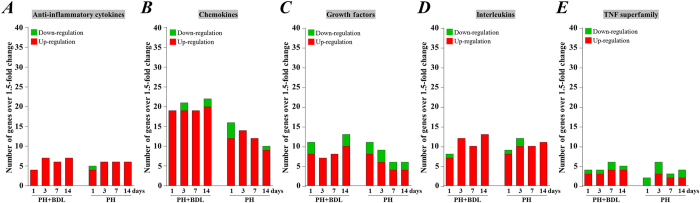
Functional categorization of genes included in the cytokine and chemokine PCR array. Based on their biological functions, these genes were categorized as anti-inflammatory cytokines (**A**), chemokines (**B**), growth factors (**C**), interleukins (**D**), and members of the TNF superfamily (**E**). We counted the number of genes that were up- and down-regulated by more than 1.5-fold in the liver tissue of mice at 1, 3, 7, and 14 days after partial hepatectomy and bile duct ligation (PH+BDL group) or partial hepatectomy alone (PH group).

Among the genes that were included in the cytokines and chemokines PCR array, we detected a very similar change in number of genes encoding anti-inflammatory cytokines (Fig. 7A) and interleukins (Fig. 7D) between the PH and PH+BDL groups, during the 14-day follow-up period. In contrast, the number of genes that were functionally related to chemokines (Fig. 7B) and growth factors (Fig. 7C) decreased over time in the PH group but remained stable in the PH+BDL group during the entire follow-up period. At 1 day after surgery, several genes of the TNF superfamily were enhanced in the PH+BDL group, but no enhancement was observed in the PH group (Fig. 7E).

## Discussion

With the increasing number of living donor liver transplantations (LDLT), biliary complications in donors have emerged as a major postoperative problem. Biliary stricture can cause cholestasis, which is one of the most common complications in patients who have undergone LDLT and is related to many liver disorders^14^. Bile duct stricture may also elevate serum aminotransferases, bilirubin, alkaline phosphatase, and gamma-glutamyl transferase levels. All of those pathological conditions can induce cell damage and eventually lead to fibrosis and cirrhosis of liver. In the patients who received LDLT, the donor liver will start to grow in size by initiating a regenerative process, including the proliferation of hepatocytes and the differentiation/maturation of hepatic progenitor cells. Although the accumulation of bile acids is well known to induce hepatocellular injury and lead to liver fibrosis, the role of cholestasis in liver regeneration and the relative mechanism remains largely unclear. By using an animal model with bile duct ligation, we explored the factors that may impair liver regeneration in the cholestatic liver.

The liver is known to initiate the regenerative process following partial hepatectomy, and bile duct ligation is one of the most common methods used to establish a cholestatic liver model. Since we were interested in pursuing the regenerative response under cholestatic conditions we did not examine the BDL only group in our current study. The rationale being that BDL is not sufficient to mount a regenerative response in the liver but rather mimics injury and damage subsequent to cholestasis^15^,^16^. Compared with the healthy liver, the cholestatic liver was smaller in size and had a lower cell proliferative activity and observable fibrotic change 14 days after partial hepatectomy, indicating insufficient regeneration. Because we performed the surgical procedures of partial hepatectomy and bile duct ligation at the same time, the “seed” for liver regeneration, including hepatocytes and hepatic progenitor cells should have been comparable in quality and quantity at the time of initiation of the regenerative process following partial hepatectomy. Therefore, we tried to identify the key “soil” factors in liver regeneration by monitoring the changes in gene expression levels in healthy and cholestatic livers after partial hepatectomy.

Transcriptional analysis is commonly used to reveal gene expression patterns and gene regulation relationships, and to identify differentially expressed genes between samples with a given disease and healthy controls^17^. Although microarrays and RNA-seq approaches are widely used for large-scale transcriptome analysis^18^, the resulting data have relative poor reliability and need to be further validated by real-time reverse transcription PCR (RT-PCR)^19^,^20^. In contrast, a pathway-focused PCR array is a gene profiling method based on the RT-PCR technique, and shows a high reliability and specificity^21^. Given that the “soil” factors should mainly contribute to the insufficient regeneration of the cholestatic liver, we selected two PCR arrays that specially focused on the inflammatory cytokines and the ECM and adhesion molecules, to identify the potential molecules associated with liver regeneration.

Many genes were up- and down-regulated by more than 1.5-fold after partial hepatectomy in either healthy or cholestatic livers. In healthy liver compared to cholestatic liver, the overall number of up- and down-regulated genes on ECM & adhesion molecules was much lower during the early phase and dramatically decreased 14 days after partial hepatectomy. Because the liver size and cell proliferative activity had nearly recovered within 14 days after partial hepatectomy in the healthy liver, many of these genes involved in the liver regenerative process may return to normal levels soon after the completion of liver regeneration. Unexpectedly, the overall number of up- and down-regulated genes encoding inflammatory cytokines and chemokines was not largely different between the cholestatic and healthy livers after partial hepatectomy, although the accumulation of bile acids is commonly considered to be an inflammatory stimulus.

We further categorized these genes according to their biological functions and found that the ECM, adhesion molecules, and transmembrane molecules were much less likely to be enhanced in the healthy liver, especially in the later phases after partial hepatectomy. Similarly, fewer inflammatory chemokines were enhanced in the healthy liver after partial hepatectomy. We also noticed that none of the genes from the TNF superfamily were up-regulated in the healthy liver 1 day after partial hepatectomy. Although we did not perform an additional interventional experiment to confirm the functional role of each gene, the enhanced expression of inflammatory chemokines and molecules involved in the ECM and adhesion should be the most likely factors impairing liver regeneration after partial hepatectomy.

The expression of Thbs1, a factor that directly induces tissue injury and hepatic fibrosis^22^, was enhanced in the cholestatic liver 3 days after partial hepatectomy. The expression of Ccl2, one of the important chemokines associated with non-alcoholic steatohepatitis and hepatic fibrosis^23^, was also increased in the cholestatic liver. Consistently with these findings from immunohistochemistry analysis, our data from the PCR array precisely showed the enhanced expression of *Thbs1* and *Ccl2* in the cholestatic liver, which also indirectly indicated the high reliability of the data from the PCR array analysis that we used in the present study. Based on our best knowledge, the upregulation of *Thbs1*has not previously been detected following BDL, although BDL treatment is known to induce the expression of *Ccl2*^24^.

This study has several limitations. First, although the primary purpose of this study was to identify the potential factors that impair liver regeneration in the cholestatic liver, it would have been better to have included another control group with BDL only because the accumulation of bile acids can induce cell damage (such as apoptosis/necrosis)^7^,^8^,^9^. However, many genes largely changed in the PH+BDL group have not previously been detected after BDL. This suggested indirectly that the procedure of partial hepatectomy should be critical for initiating the regeneration of liver. Second, we did not identify the key molecules by additional experiments, such as an *in vitro* functional analysis, and we are unable to present all of the PCR array data in detail, owing to a patent application. The changes in liver weight and cell proliferation over time indicated poor regeneration of the cholestatic liver beyond 7 days after hepatectomy. However, more parameters, such as cell apoptosis and necrosis, and even longer-term follow-up, may be needed to carefully evaluate the liver regeneration capacity, especially for the cholestatic liver.

In conclusion, data from the present study showed that many factors, especially extracellular matrix components and inflammatory chemokines are likely involved in liver damage and the initiation of the regenerative process. Continuous enhancement of extracellular matrix and inflammatory chemokines may negatively regulate liver regeneration, but further experiments with interventional treatments are required to confirm the key factor(s).

## Materials and Methods

### Animal models

We used 8- to 10-week-old male C57BL/6 mice (SLC, Japan) for this study. All of the experiments received approval from the Institutional Animal Care and Use Committee of Nagasaki University (No. 1108120943), and the experiments were performed in accordance with institutional and national guidelines.

A total of 48 mice were randomly subjected to a 70% partial hepatectomy and bile duct ligation (PH+BDL group, n = 16), a 70% partial hepatectomy only (PH group, n = 16), or a sham operation with laparotomy (Sham group, n = 16). All of the surgical procedures were performed according to a previous study^25^,^26^. Briefly, after general anaesthesia, laparotomy was performed by using midline abdominal skin and muscle incisions. For the PH+BDL group, the common bile duct was exposed and ligated with 6–0 silk sutures, which was followed by a 70% partial hepatectomy. For the PH group, we only exposed the common bile duct (without ligation), and then performed a 70% partial hepatectomy. Four mice in each group were sacrificed at 1, 3, 7 and 14 days after treatment, respectively. The body weight was measured before sacrifice. The liver tissue was extracted and weighed. The ratio of liver to body weight was calculated to evaluate liver regeneration ability. Liver tissues were collected for the subsequent experiments.

### Histological analyses

Half of the liver tissue from each mouse was used for histological analyses. Briefly, liver tissue were fixed in 4% paraformaldehyde phosphate buffer solution for 12 hours, dehydrated with high concentrations of ethanol, embedded in paraffin, and then cut into 5-μm thick sections. The sections were stained with hematoxylin-eosin (HE) and Masson’s trichrome. All of the staining procedures were performed based on standard processes. Five fields were randomly selected for quantitative measurement of the fibrotic area by using Winroof, and the averages of the fibrotic area were used for statistical analysis.

To evaluate liver regeneration, immunohistochemistry analysis was performed to detect the cell proliferation antigen Ki67^27^, a protein presenting in all of the active phases of the cell cycle (G_1_, S, G_2_, and mitosis) but absent from resting cells (G_0_). Immunohistochemistry analysis was also performed to confirm the expression of several factors that previously were shown to be associated with inflammation and hepatic fibrosis, including thrombospondin 1 (Thbs1) and the chemokine (C-C motif) ligand 2 (Ccl2)^22^,^23^. Positive staining was visualized using 3′3-diaminobenzidine^27^. More than 5 fields from at least 2 slices of each tissue sample were randomly selected for semi-quantification, and the averages were used for statistical analysis.

### Pathway-focused PCR array

To search the factors associated with liver regeneration, a pathway-focused array analysis was performed. Briefly, total RNA was isolated from half of the freshly collected liver tissues from each mouse by using an RNeasy Mini Kit (Qiagen). We used 1 mg of total RNA from each sample and pooled all of the 4 RNA samples at the same time point in each group to generate cDNA using the RT2 First Strand Kit (SABiosciences). A mouse extracellular matrix and adhesion molecule PCR array and a cytokine and chemokine PCR array, each containing a total of 84 genes, were used according to the manufacturer’s instructions (SABiosciences)^28^,^29^,^30^. All of the data were normalized to the expression of housekeeping genes, and the expression level for each gene was quantified based on the cycle threshold (Ct). Data analysis was performed with a web-based analysis program (SABiosciences, http://pcrdataanalysis.sabiosciences.com/pcr/arrayanalysis.php). We selected the genes that were up- and down-regulated by more than 1.5-fold for further categorization analysis according to their biological function.

### Statistical analyses

All of the results are presented as means ± SD. The statistical significance was determined by a one-way analysis of variance followed by the Bonferroni post hoc test (Dr. SPSS II, Chicago, IL). Differences were considered significant at *p* < 0.05.

## Additional Information

**How to cite this article**: Zhang, S. *et al.* Up-regulated extracellular matrix components and inflammatory chemokines may impair the regeneration of cholestatic liver. *Sci. Rep.* **6**, 26540; doi: 10.1038/srep26540 (2016).
